# Rethinking Energy Availability from Conceptual Models to Applied Practice: A Narrative Review

**DOI:** 10.3390/nu18030379

**Published:** 2026-01-23

**Authors:** Sergio Espinar, Marina A. Sánchez-Fernández, Juan J. Martin-Olmedo, Marcos Rueda-Córdoba, Lucas Jurado-Fasoli

**Affiliations:** 1 Faculty of Health Sciences, Catholic University of Murcia, 30107 Murcia, Spain; 2 Sport and Health University Research Institute (iMUDS), University of Granada, 18071 Granada, Spain; 3 Department of Physiology, Faculty of Medicine, Sport and Health University Research Institute (iMUDS), University of Granada, 18071 Granada, Spain; 4 Centro de Investigación Biomédica en Red Fisiopatología de la Obesidad y Nutrición (CIBERobn), Instituto de Salud Carlos III, 28029 Madrid, Spain; 5 Instituto de Investigación Biosanitaria ibs.GRANADA, 18012 Granada, Spain

**Keywords:** endocrine adaptations, metabolic suppression, female athlete triad, energy deficiency, sports nutrition

## Abstract

**Background/Objectives**: Energy availability (EA), defined as the dietary energy remaining after exercise energy expenditure (EEE), is a central determinant of both health and performance in athletes. Chronic insufficient EA leads to low energy availability (LEA), which is an underlying mechanism of Relative Energy Deficiency in Sport (REDs). This narrative review critically explores the conceptual evolution of EA and LEA, summarizes current physiological evidence, and discusses methodological and practical challenges in their assessment and application in free-living athletes. **Methods**: Evidence from experimental and observational studies was reviewed to describe the hormonal, metabolic, and performance outcomes associated with LEA. Screening tools, including the Low Energy Availability in Females Questionnaire (LEAF-Q) and the Low Energy Availability in Males Questionnaire (LEAM-Q), were also evaluated for their validity and applicability in different sports contexts. **Results**: LEA is associated with alterations in thyroid and reproductive hormones, which, in turn, contribute to reduced resting metabolic rate, lower bone mineral density, and delayed recovery. While screening questionnaires can help identify athletes at risk, their accuracy varies by sport and individual characteristics. Incorporating hormonal and metabolic biomarkers provides a more direct and sensitive method for detecting physiological stress. Measuring dietary intake, EEE, endocrine balance and body composition in real-world settings remains a major methodological challenge. Combining hormonal, metabolic, and behavioral indicators may improve the identification of athletes experiencing LEA. **Conclusions**: EA plays a central role in the interaction between nutrition, exercise, and athlete health, but methodological limitations in its assessment may compromise accurate diagnosis. Improving measurement techniques and adopting integrated monitoring strategies are essential to improve early detection, guide individualized nutrition, and prevent RED-related health and performance impairments.

## 1. Introduction

Meeting the energetic demands of intense training presents a constant challenge for athletes and is a key determinant of both health and performance [1]. When this balance is not matched, adaptive mechanisms are triggered to conserve energy, primarily through endocrine and metabolic adjustments that reprioritize essential physiological functions [2,3]. To characterize these physiological interactions, Loucks introduced the concept of energy availability (EA), defined as the amount of dietary energy remaining for essential physiological processes after accounting for the energetic cost of exercise [4], and expressed relative to fat-free mass (FFM) [5].

Although closely related, EA and energy balance represent distinct constructs. Energy balance describes the long-term relationship between energy intake (EI) and total daily energy expenditure (TDEE), typically inferred from long-term changes in body mass [6,7]. In contrast, EA reflects energetic sufficiency, indicating whether the EI adequately supports ongoing biological functions once exercise demands are met [6]. Consequently, an athlete may maintain a stable body weight while experiencing substantial hormonal and metabolic disturbances due to insufficient EA. Loucks and Thuma demonstrated this distinction by showing that reducing EA from 30 to 20 kcal/kg FFM/day suppressed luteinizing hormone (LH) pulsatility, a sign of energy inadequacy, despite no significant differences in body weight between conditions [8].

Such a distinction is particularly relevant in sports emphasizing high training volumes or leanness, where the margin for maintaining energy equilibrium is narrow [9]. Under these conditions, dietary intake may fail to fully meet the energetic demands of training, leading to periods of low energy availability (LEA) [10].

LEA is typically defined as an EA < 30 kcal/kg FFM/day, a threshold below which compensatory endocrine and metabolic adaptations may occur [7,11]. However, this value should not be interpreted as a rigid diagnostic criterion. Derived from controlled laboratory studies, it may not fully capture the complexity and variability of responses observed in free-living athletic populations [12,13,14]. Hänisch et al. (2025) highlight considerable inter-individual variability in the physiological responses to reduced EA, underscoring the need to interpret EA on a spectrum rather than applying fixed cut-offs [15].

Although endocrine assessments are frequently employed in LEA research, there is currently no consensus on a minimal biomarker panel suitable for routine practice, largely due to marked inter-individual variability and the influence of multiple non-LEA-related stressors. These limitations have prompted the development of integrative monitoring frameworks, such as the Athlete Health and Readiness Checklist (AHaRC), which view endocrine markers as indicators of physiological adaptation and maladaptation rather than standalone diagnostic criteria, to be interpreted within a broader longitudinal and contextual assessment of athlete health.

Given the limitations of isolated EA metrics, the variability of endocrine responses, and the coexistence of multiple conceptual frameworks, a comprehensive synthesis is warranted. This narrative review aims to integrate current evidence on EA and LEA, discuss key methodological and physiological challenges, and provide a coherent framework to support early identification and management of LEA in athletes.

## 2. Methodology

This review prioritized recent experimental and field-based studies while acknowledging the heterogeneity of methodologies and outcome definitions across the literature. The methodology followed an updated search strategy adapted from previous reviews on the topic. A comprehensive literature search was carried out using electronic databases, including PubMed, Web of Science and Medline, focusing on literature published between January 2020 and September 2025. Search terms included combinations of the following keywords: “low energy availability”, “LEA”, “energy deficiency”, “REDs” (relative energy deficiency in sport), “female athlete triad”, “exercise energy expenditure”, “resting metabolic rate”, and “performance impairments”.

## 3. Conceptual Fundamentals of Energy Availability

Early investigations into EA sought to explain why amenorrheic athletes exhibited disruptions in reproductive and stress-related hormones compared to their eumenorrheic or sedentary counterparts [16]. Initial observational investigations into the health consequences of intensive training revealed consistent associations between disordered eating (DE), menstrual dysfunction, and impaired bone health in athletes [16,17,18,19].

Researchers subsequently questioned whether these disturbances resulted from the physical stress of exercise itself or from an energetic imbalance between EI and EEE [20]. Pioneering experimental studies by Professor Anne Loucks and colleagues demonstrated that restricting EI relative to EEE suppressed metabolic and reproductive hormones, reduced bone formation, and increased bone resorption [8,21,22]. This body of evidence established that it was insufficient EA, rather than exercise stress alone, that triggered these physiological adaptations, clarifying early on the central etiological role of EA in athlete health.

In 1992, the American College of Sports Medicine published the first Position Stand on the Female Athlete Triad [23], defining the syndrome as the coexistence of three interrelated conditions: DE, menstrual disturbances, and osteoporosis. However, dissatisfaction soon emerged because this model captured only the most severe clinical manifestations. De Souza and Williams later proposed a broader, spectrum-based model that recognized subclinical presentations across all three components [24]. This perspective redefined the Triad as a continuum of conditions characterized by LEA (with or without DE), functional hypothalamic amenorrhea (FHA), and osteoporosis [25]. However, it was later recognized that not all diagnostic criteria needed to be present, given substantial variability by sport type, highlighting the potential for underdiagnosis [26].

To address these limitations, the International Olympic Committee (IOC) introduced in 2014 the concept of Relative Energy Deficiency in Sport (REDs), positioning LEA as the core etiological factor [27]. This framework emphasized LEA as a central factor underlying disruptions in multiple physiological systems, including reproductive, endocrine, metabolic, cardiovascular, gastrointestinal, and immune functions, ultimately affecting both athletic performance and overall health. Subsequent IOC consensus statements in 2018 [28] and 2023 [29] have further refined the REDs model, supporting its clinical application across diverse athletic contexts.

Despite its widespread adoption, the REDs framework remains subject to critical debate. Several authors (including Jeukendrup, Areta, Koehler and colleagues) argued that REDs should be conceptualized primarily as a theoretical construct rather than a fully validated clinical syndrome [30]. They highlight that much of the foundational evidence supporting REDs derives from very small, short-term experimental studies, making strong causal inferences difficult. Moreover, many features commonly attributed to REDs (e.g., fatigue, menstrual irregularities, low bone mineral density, impaired immunity) are non-specific and may arise from training load, stress, or sleep disruption rather than energy deficiency itself. From this perspective, accurately diagnosing REDs is challenging because LEA cannot be measured with high precision in free-living athletes.

In response to these concerns, Areta and colleagues have proposed the AHaRC as a broader, multidimensional framework [31]. Rather than centering the diagnosis exclusively on LEA, the AHaRC compiles metrics related to nutrition, training load, recovery, psychological status, and clinical symptoms to understand the overall readiness and health status of the athlete.

Conversely, Mountjoy and colleagues maintain that REDs is a valid clinical syndrome, drawing upon extensive observational data, accumulated clinical experience, and the use of validated diagnostic tools such as the IOC REDs CAT2 [32,33]. They argue that, although long-term randomized trials are neither feasible nor ethical, decades of observational data and clinical case series consistently document clusters of endocrine suppression, impaired bone health, metabolic downregulation, and performance decrements in athletes exposed to sustained energetic deficits. According to this view, the absence of ideal experimental evidence does not negate the clinical reality of REDs. Rather than invalidating the model, these limitations highlight the need for sport-specific reference values and interdisciplinary management strategies.

Taken together, these perspectives highlight that, while REDs offers a valuable clinical description of the multisystem consequences associated with sustained LEA, its empirical foundation remains limited by small sample sizes, short-term interventions and the difficulty of measuring EA with precision in free-living athletes. These constraints reduce its diagnostic certainty and risk stratification accuracy in free-living athletes. In contrast, the AHaRC framework provides a broader and more pragmatic structure for athlete health monitoring, integrating nutritional, psychological, training-load and recovery indicators without relying on precise EA quantification. For these reasons, in this review, we adopt AHaRC as the more operationally robust framework for day-to-day athlete management, while viewing REDs as a useful (but currently incomplete) clinical model that requires further empirical validation.

## 4. Screening Tools

Accurately identifying athletes at risk of LEA and its associated health consequences remains a major challenge in both research and applied practice. Direct quantification of EA requires precise measurement of EI, EEE and body composition, an approach rarely feasible outside controlled laboratory settings due to its considerable cost and the specialized technical resources required. Consequently, screening questionnaires have been proposed as practical, first-line tools to capture symptomatic clusters associated with LEA. Their use, however, must recognize population-specific limitations and the absence of robust validation across sport types and age groups.

### 4.1. Low Energy Availability in Females Questionnaire

The Low Energy Availability in Females Questionnaire (LEAF-Q), developed by Melin et al. in 2014, was the first validated instrument designed to screen for LEA and the Female Athlete Triad in women [34]. Initially tested in endurance athletes and dancers (groups known for high susceptibility to energy deficiency) [34,35], this questionnaire consists of 25 items covering three main domains: menstrual function, gastrointestinal symptoms, and injury history. A total score of ≥8 classifies athletes as “at risk”. This approach has been widely adopted in both research and applied settings, being referenced in the IOC REDs CAT2 as a recommended screening tool [29].

In its original validation, the LEAF-Q demonstrated sensitivity of ~78% and specificity near 90% in endurance athletes [34]. While these metrics support its utility, they cannot be generalized to all sports, as subsequent studies have shown variable accuracy in team-sport, aesthetic and strength/power athletes [36,37]. Moreover, false positives commonly arise when symptoms stem from conditions not directly linked to LEA, such as gastrointestinal disorders or polycystic ovary syndrome [38,39], while false negatives occur frequently in athletes using hormonal contraceptives, which mask menstrual irregularity, one of the most sensitive physiological markers of LEA [40,41,42]. Therefore, LEAF-Q results should never be interpreted in isolation. Clinical assessment, training, nutrition history, and endocrine evaluations are essential for a comprehensive interpretation.

### 4.2. Androgen Deficiency in Aging Males Questionnaire

Before male-specific tools were developed, the ADAM-Q was frequently used in sports research to explore potential androgen deficiency in male athletes [43,44]. However, this questionnaire was originally designed for middle-aged clinical populations and shows poor specificity in younger, highly active men [45]. Core symptoms such as fatigue, low energy and reduced vitality are common in training environments for reasons unrelated to suppressed androgen production. In its original validation, 37% of healthy eugonadal men screened “positive”, demonstrating an unacceptably high false-positive rate [45]. Furthermore, the ADAM-Q has never been validated against hormonal or metabolic markers of LEA in athletes, and its established cut-off thresholds do not align with the natural hormonal variability characteristic of training populations. Given these limitations, the ADAM-Q is not recommended as an LEA-specific screening tool in athletic populations.

### 4.3. Low Energy Availability in Males Questionnaire

Recognizing the previous gap, the Low Energy Availability in Males Questionnaire (LEAM-Q) was recently developed as the first male-specific instrument for screening LEA in male athletes. The questionnaire was initially designed based on expert consensus and existing LEA frameworks, and later validated in a cohort of more than 300 elite and sub-elite male athletes, primarily from endurance and weight-sensitive sports [14]. Item selection was informed by expert consensus and by validated questionnaires addressing physiological domains potentially affected by chronic energy deficiency, including fatigue, illness, dizziness, gastrointestinal function, and libido [14].

Initial applications provide encouraging evidence of construct validity. Athletes classified as “at risk” often demonstrate co-occurring features such as disordered eating, compulsive exercise tendencies and training-related preoccupation [42,46]. Importantly, LEAM-Q scores show no meaningful association with circulating testosterone [42,44], reinforcing emerging consensus that single hormonal measurements are insufficient for diagnosing or excluding LEA in male athletes.

Nevertheless, its current validation is limited to endurance and leanness-focused sports, and no universal cut-off score has been established, restricting its generalizability to other sport disciplines. Thus, while the LEAM-Q constitutes a critical step toward nuanced male screening, its outputs must be supplemented by clinical judgment and objective physiological markers.

### 4.4. Methodological Considerations Across Screening Tools

While screening questionnaires offer practical, low-cost solutions for initial LEA risk identification, they share inherent methodological limitations that constrain their diagnostic precision. As fully self-reported instruments, they are susceptible to recall bias, social desirability and misinterpretation [47,48,49], challenges that become particularly salient when assessing sensitive domains such as menstrual function, libido, psychological symptoms or disordered eating [50,51]. Additionally, many queried symptoms (such as fatigue, low mood, gastrointestinal discomfort, irregular menses or reduced libido) are non-specific and may reflect high training loads, illness, psychological stress or contraceptive use rather than LEA itself. This non-specificity explains the false positives and false negatives consistently observed in evaluations of the LEAF-Q and LEAM-Q [36,37,40,42].

A further limitation is the restricted scope of validation studies, which largely derive from endurance and weight-sensitive sports, limiting generalizability to team-sport, sprint/power and intermittent disciplines. For male athletes, the LEAM-Q lacks an established universal cut-off and its diagnostic performance outside endurance sports remains unknown.

In summary, LEA questionnaires should therefore be understood primarily as risk stratification tools rather than diagnostic instruments, meaning that athletes flagged as “at risk” require follow-up through clinical evaluation, nutritional assessment and objective physiological indicators. From an applied standpoint, LEAF-Q and LEAM-Q are best administered at baseline and again during periods of weight loss, increased training volume, or whenever clinical suspicion of LEA arises. Questionnaire results should be interpreted in combination with a panel of blood biomarkers associated with LEA to strengthen diagnostic confidence. In cases where scores are borderline or ambiguous, repeating the questionnaire, reviewing recent training and nutritional patterns, and temporarily increasing monitoring frequency are recommended before making dietary adjustments.

## 5. Metabolic and Physiological Markers of Low Energy Availability

Over the past two decades, research has increasingly examined the physiological mechanisms through which LEA (especially <30 kcal/kg FFM/day) affects hormonal regulation and metabolic function in athletes. These endocrine and metabolic adaptations not only indicate energy stress but also have direct implications for recovery, performance, and training adaptations [52,53]. The thyroid, reproductive and metabolic axes appear to be among the most sensitive systems to LEA [54,55], with alterations that may persist throughout competitive seasons [56,57]. Given their consistency across sexes, sports, and experimental conditions, these markers have emerged as practical indicators of LEA in applied athletic settings [58] though their expression is subject to substantial inter-individual and sport-specific variability.

Although research on LEA frequently relies on endocrine assessments, there is currently no consensus on a minimal biomarker panel suitable for routine practice, largely due to inter-individual variability and the influence of non-LEA-related factors. Consequently, endocrine markers should not be interpreted in isolation, but rather integrated longitudinally with clinical and behavioral data.

### 5.1. Hypothalamic–Pituitary–Thyroid Axis and Low Energy Availability

Alterations in the thyroid axis represent one of the most reproducible endocrine responses to LEA, particularly evident in the suppression of circulating triiodothyronine (T_3_) concentrations [7]. As a central regulator of resting metabolic rate (RMR), mitochondrial function, and thermogenesis [59,60], T_3_ serves as a sensitive integrator of energetic status.

Loucks et al. demonstrated that circulating T_3_ levels begin to decline when EA acutely drops to between 19.0 and 25.0 kcal/kg FFM/d, even though thyroxine (T_4_) remains unchanged [61]. Notably, when EA was ≤10 kcal/kg FFM/day, T_4_ levels rose slightly, potentially reflecting a compensatory reduction in T_4_-to-T_3_ conversion as part of an energy-conserving adaptation. These findings align with other controlled trials showing that caloric restriction typically induces a 10–25% decrease in circulating T_3_, whereas T_4_ concentrations tend to remain stable [62,63,64]. Cross-sectional research further supports these observations, reporting lower T_3_ levels both in female athletes with FHA and male endurance athletes chronically exposed to energy deficits [25,65,66,67].

Collectively, these data suggest that reductions in T_3_ are among the earliest and most reliable endocrine markers of LEA. Although T_4_ concentrations usually remain stable in the short term, prolonged or severe LEA can gradually affect T_4_ as well, amplifying metabolic suppression [8]. This downregulation represents an adaptive physiological mechanism that lowers basal EE and supports metabolic homeostasis during sustained energy restriction.

### 5.2. Hypothalamus–Hypophysis–Gonadal Axis and Low Energy Availability

The hypothalamic–pituitary–gonadal (HPG) axis plays a central role in coordinating reproductive and metabolic homeostasis. Under LEA conditions, pulsatile gonadotropin-releasing hormone (GnRH) secretion is suppressed, leading to reduced LH and follicle-stimulating hormone (FSH) release, with subsequent decreases in sex steroid production (estradiol in women and testosterone in men) [68]. Such endocrine alterations are generally interpreted as adaptive reallocations of metabolic resources toward essential processes when energetic supply becomes constrained [69,70,71].

#### 5.2.1. Functional Hypothalamic Amenorrhea (FHA)

In female athletes, suppression of GnRH pulsatility is a well-established response to sustained LEA, leading to downstream inhibition of ovarian function [72]. Classic studies by Loucks and colleagues demonstrated that EA < 30 kcal/kg FFM/day decreases LH pulsatility, a sign of hypothalamic suppression [8]. Complementing these threshold-based observations, population-level analyses indicate that menstrual disturbances occur across a continuum of EA values, with each 1 kcal/kg FFM/d reduction increasing the odds of menstrual dysfunction by ~9% [73]. These findings underscore substantial inter-individual variability in sensitivity to energetic stress, supporting the view that reproductive suppression is not restricted to a fixed EA cut-point but may emerge under a wider range of energetic conditions [74].

FHA carries important consequences for bone health. Chronic hypoestrogenism impairs bone formation and accelerates bone resorption, placing affected athletes at increased risk of reduced bone mineral density and bone stress injuries [75,76,77,78,79,80,81]. Longitudinal studies show that women with FHA have up to 4.5-fold greater bone injury risk than eumenorrheic athletes, while adolescent female athletes with LEA exhibit compromised peak BMD [82,83,84].

Beyond reproductive and skeletal outcomes, LEA-related alterations in T_3_, estradiol and cortisol have been linked to impaired metabolic efficiency, reduced muscle protein synthesis and suboptimal neuromuscular recovery [85,86,87,88]. Athletes with FHA have shown lower strength and power capacities [89], as well as higher prevalence of fatigue, mood disturbances and sleep disruption, likely reflecting broader neuroendocrine strain [90]. Taken together, FHA represents an integrated multisystem response to sustained energetic deficit, with implications for both health and sport performance.

#### 5.2.2. Male Hypogonadism

Reproductive suppression in male athletes follows a distinct pattern. Unlike women, men lack an overt clinical marker such as menstrual status; therefore, hypogonadism may develop with few or no outward symptoms despite measurable alterations within the HPG axis. Prolonged LEA can reduce GnRH pulsatility and LH secretion, ultimately lowering testicular steroid production [91].

This condition, known as “Exercise Hypogonadal Male Condition (EHMC)”, is classified within the REDs framework as a functional and reversible downregulation of the reproductive axis [29,92].

Rather than reflecting a pathological failure of the gonads, EHMC represents a functional, reversible downregulation of the HPG axis [93,94,95,96]. Experimental work indicates that reductions in LH pulsatility can occur within 48 h of severe energetic deficit [97], highlighting the acute sensitivity of the male HPG axis to LEA. Evidence from endurance athletes and military cohorts further suggests that these alterations reverse once adequate energy availability is restored [94,98]. Although primarily adaptive from an energy-conservation perspective, suppressed anabolic signaling may compromise muscle protein synthesis, bone remodeling and tissue repair, collectively increasing injury risk and impairing training adaptations [85,99,100,101].

The endocrine suppression associated with LEA exerts wider systemic effects that extend beyond the musculoskeletal system. Long-term neuroendocrine alterations (including elevated cortisol and reduced anabolic hormones) can negatively affect mood, motivation, and sleep regulation, contributing to fatigue, irritability, and depressive symptoms frequently reported in REDs contexts [46,102,103,104,105].

In summary, reproductive alterations in both women and men represent coordinated, energetically driven adjustments across the HPG axis. While the mechanisms differ by sex, both FHA and exercise-related hypogonadism emerge as components of a broader adaptive response to LEA, with potential consequences for physiological function, health and sport performance.

### 5.3. Metabolic Adaptations to Low Energy Availability

LEA also disrupts the broader neuroendocrine network responsible for sensing and communicating energetic status. Leptin, insulin, and IGF-1 are pivotal hormones linking EI, body composition, and metabolic function. Their collective response reflects an orchestrated regulatory mechanism through which the body prioritizes survival and energy conservation over growth and performance.

#### 5.3.1. Leptin

Leptin acts as a key signal linking peripheral energy stores to central neuroendocrine regulation. It reflects both long-term fat stores and short-term fluctuations in EA [106,107,108], and its circulating levels decline proportionally to the energy deficit, independently of body mass changes [109,110]. By modulating hypothalamic control of GnRH pulsatility, leptin influences reproductive regulation [111]; reduced leptin during sustained energy deficiency contributes to hypothalamic downregulation and the reproductive suppression characteristic of REDs [29].

Leptin levels differ by sex, with women consistently exhibiting higher concentrations than men. This difference is largely explained by greater overall adiposity and estrogen-driven leptin synthesis [112,113]. Such sex-specific regulation may help explain the greater susceptibility of female athletes to FHA [29]. Laughlin and Yen reported that both eumenorrheic and amenorrheic endurance athletes displayed approximately threefold lower 24 h serum leptin concentrations than sedentary controls, although only eumenorrheic athletes maintained a normal diurnal rhythm of leptin secretion [114].

Experimental evidence further supports leptin’s causal role in reproductive restoration. Administration of recombinant leptin in women with FHA has been shown to restore gonadotropin secretion, increase estradiol concentrations, and reinstate menstrual function [115]. These findings identify leptin as a permissive signal of energy sufficiency, coordinating metabolic and reproductive axes in response to nutritional status. Accordingly, alterations in leptin should be interpreted as part of a wider endocrine signature of energetic strain rather than as an isolated biomarker of LEA.

#### 5.3.2. Insulin and Hepatic–Endocrine Adaptations to Low Energy Availability

LEA is consistently associated with suppressed fasting insulin and a broader shift toward an energy-conserving endocrine profile in female athletes [116]. Comparable endocrine adaptations have also been observed in healthy men exposed to controlled energy restriction. In a military cohort subjected to a severe caloric deficit of approximately 1200 kcal/day, accompanied by high physical demands, participants developed a transient, subclinical hypogonadism characterized by reductions in insulin, T_3_, insulin-like growth factor 1 (IGF-1), and testosterone. These alterations returned to baseline after a period of controlled refeeding, confirming that the endocrine suppression was primarily driven by the energy deficit rather than by exercise stress alone [98].

Reduced insulin levels during LEA also promote hepatic upregulation of sex hormone-binding globulin (SHBG), thereby reducing free testosterone without altering total testosterone [117]. These changes likely reflect an adaptive endocrine response to LEA rather than a purely pathological dysfunction. Insulin further regulates substrate partitioning by stimulating glucose uptake via GLUT4 translocation and activating glycogen synthase [118,119]. When insulin remains chronically low, glycogen resynthesis after exercise is impaired, shifting metabolism toward greater fat oxidation and reducing the capacity for high-intensity, glycolytic performance [120,121].

Although women oxidize proportionally less carbohydrate and more fat at comparable exercise intensities, maintaining adequate glycogen stores remains essential for optimal performance. Walker et al. (2000) demonstrated that carbohydrate loading increases muscle glycogen content and prolongs time to fatigue in well-trained female athletes, underscoring the importance of glycogen availability for both sexes [122]. From a practical standpoint, the combination of low energy and low carbohydrate availability increases the likelihood of beginning subsequent training sessions with suboptimal glycogen stores, thereby amplifying typical symptoms of energy deficiency such as early fatigue, slower recovery, and reduced training quality [123].

From a performance perspective, the effects of LEA are heterogeneous and context-dependent. Current evidence indicates that LEA does not consistently impair maximal aerobic capacity (VO_2_max), peak power output, or anaerobic threshold, particularly during short-term or moderate energy restriction [88]. Instead, performance decrements are more frequently observed in measures related to training tolerance, fatigue, neuromuscular function, and explosive power, often arising through indirect mechanisms such as impaired recovery, reduced training quality, or low carbohydrate availability rather than uniform declines in maximal performance markers.

#### 5.3.3. RMR and RMR Ratio

RMR constitutes the largest component of TDEE and represents the energy needed to sustain vital physiological functions at rest. RMR is highly responsive to energetic status, and in athletes, LEA can induce a reduction in RMR that conserves energy during chronic underfueling, but further exacerbates the overall energetic deficit [2,124]. Therefore, the assessment of RMR through indirect calorimetry has been proposed as a marker of an athlete’s energy status. Persistently low RMR values may reflect the physiological adaptations associated with REDs, including hormonal disturbances, impaired bone metabolism, and blunted training adaptations [10,52].

To enhance interpretability, researchers have proposed the RMR ratio, defined as measured RMR divided by predicted RMR. Values below 0.90 are typically interpreted as indicative of metabolic suppression and are often used as a surrogate marker of chronic LEA [125,126]. Nevertheless, evidence remains inconsistent. Some studies have reported that LEA is associated with a reduced RMRratio [125,127], while others have found no significant relationship between these variables [128,129]. This heterogeneity suggests that metabolic suppression does not occur uniformly across individuals, and that reductions in RMR or RMRratio may reflect adaptive physiological downregulation rather than a direct quantitative marker of EA. Indeed, some athletes display metabolic suppression despite apparently adequate EI, whereas others maintain normal RMR despite confirmed LEA, underscoring the limitations of relying on RMR-based markers in isolation.

Collectively, these findings highlight that RMR and RMRratio should be interpreted as downstream or adaptive responses to energetic stress rather than primary diagnostic indicators, and that their clinical relevance is substantially enhanced only when interpreted alongside endocrine markers of energy deficiency.

## 6. Methodological Challenges in the Assessment of Energy Availability

**Figure 1:**
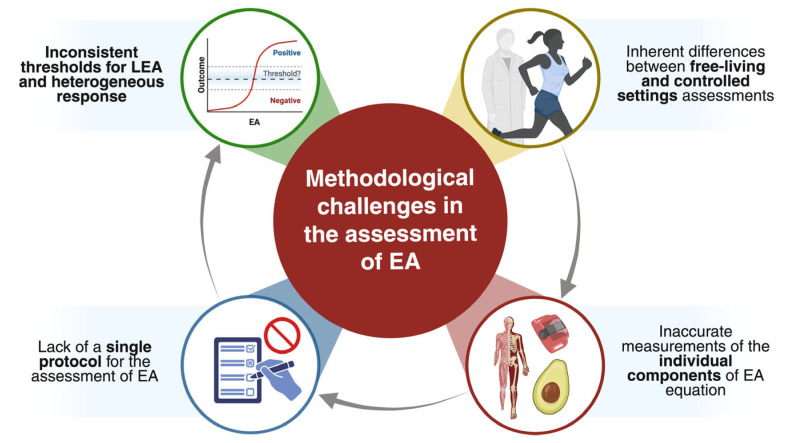
The accurate quantification of EA in athletes is limited by several methodological constraints. These include the absence of a standardized assessment protocol, inaccuracies in the measurement of individual components of the EA equation (energy intake, exercise energy expenditure, and fat-free mass), and the inherent differences between controlled laboratory conditions and free-living athletic environments. Additionally, inconsistencies in defining the threshold for low energy availability (LEA) and the wide interindividual variability in physiological responses could affect the interpretation and comparison of findings across studies.

The quantification of EA remains one of the major methodological challenges in this field. Accurate measurement requires the accurate assessment of multiple dynamic components (EI, EEE, and FFM), yet each of these components is subject to considerable error in free-living athletic environments. As a result, inconsistencies in measurement protocols, differences between laboratory and real-world conditions, and substantial inter-individual variability have contributed to the absence of universally accepted diagnostic thresholds for LEA (Figure 1). These constraints make EA an imprecise construct when applied outside controlled environments and necessitate cautious interpretation of calculated values.

In most studies, EI is estimated using prospective dietary records or 24 h recalls, which rely on self-reported intake over several consecutive days [82,130,131,132]. Although these methods are valid when properly implemented, their accuracy depends heavily on participant compliance and portion-size estimation. Factors such as underreporting, omission of snacks, and social desirability bias frequently lead to under- or overestimation of EI and, consequently, to inaccurate EA calculations [133]. On average, self-reported food records typically underestimate EI by 5–21% compared to actual intake [134]. This systematic underestimation leads to considerable uncertainty in EA calculations and may explain why field studies frequently report weak or inconsistent associations between EA and metabolic, hormonal, or menstrual outcomes [66,135].

Similar limitations exist when estimating EEE. Most research uses accelerometry [132,136,137], heart rate monitors [130,138], or activity logs, such as the Bouchard Activity Record [131,139,140]. These methods tend to underestimate the energetic cost of high-intensity, intermittent, or resistance-based exercise because device algorithms are typically calibrated for steady-state aerobic activity [141]. Although the doubly labeled water method provides an accurate measure of TDEE, it is rarely used in sports research because it cannot distinguish EEE from other activity components and is limited by high cost, logistical complexity, and reduced feasibility in free-living athletic settings. Consequently, there is currently no consensus on the most valid or standardized approach for determining EEE in free-living conditions.

Another methodological limitation is the lack of assessment of physical activity performed outside structured training sessions. In free-living athletes, non-exercise activity thermogenesis (NEAT) represents an additional and often overlooked source of variability in TDEE. Moreover, EA fluctuates within the day depending on the timing of EI relative to training, further complicating the interpretation of daily EA estimates. This variability is not accounted for in traditional EA calculations, as the classical EA equation was derived from controlled laboratory studies where physical activity remained unchanged [22,142], thereby not capturing the variability of real-world athletic environments. For instance, the magnitude of error introduced when estimating EE can substantially alter the calculated EA. Indeed, recent work has shown that replacing traditional EEE with a more comprehensive Activity-induced Energy Expenditure (AEE) (which incorporates both structured exercise and NEAT ) can markedly reduce EA values. Taguchi & Manore (2022) demonstrated that recalculating EA using AEE instead of EEE in free-living athletes decreased estimated EA from ~32 to ~20 kcal/kg FFM/day, a reduction large enough to shift an athlete’s classification from adequate EA to LEA [143].

This alternative approach to recalculating EA using AEE becomes particularly relevant in free-living athletes because NEAT represents a substantial and highly variable component of TDEE. Classic work by Levine and colleagues showed that NEAT can differ by up to approximately 2000 kilocalories per day between individuals of similar body size [144], illustrating the magnitude of variability that can occur independently of structured training. Despite its importance, NEAT is rarely quantified in applied research, and this omission could explain interindividual differences in physiological adaptations to energetic stress.

Recent technological developments may also help reduce some of the methodological uncertainty inherent in EA assessment. Multi-sensor wearable devices that integrate accelerometry, heart-rate-derived metrics, and movement signatures have shown improved accuracy in estimating activity-induced energy expenditure compared with traditional single-sensor approaches [145]. Similarly, digital dietary logging systems combined with machine-learning algorithms for food recognition and portion-size estimation are emerging as promising tools to complement or partially replace self-reported intake, which is frequently affected by systematic underreporting [146,147]. Although these technologies offer considerable potential to enhance the precision of EI and EEE estimation in free-living athletes, their validation in sport-specific contexts remains limited, and standardized protocols for their implementation have not yet been established.

Importantly, error is not confined to EI and EEE; the denominator, FFM, also contributes substantially to variability in EA calculations. Accurate assessment of FFM is therefore equally important, as EA is normalized to this value. Dual-energy X-ray absorptiometry (DXA) is commonly the most used method for assessing body composition and FFM in athletes. However, alternative techniques such as bioelectrical impedance analysis (BIA) [137] or skinfold measurements [136,140] are frequently employed in field settings due to their accessibility and lower cost. These methods are not interchangeable, and there is no consensus on the optimal approach. Compared with DXA, BIA, and skinfold methods typically underestimate fat mass and overestimate lean mass, introducing systematic bias in FFM assessment [148,149,150].

This methodological variability further limits the comparability of EA values across studies and complicates the interpretation of findings across studies. A structured summary of current approaches, their main limitations, and practical recommendations for improving EA assessment in applied settings (Table 1).

Considering all these limitations, from inaccurate EI reporting, variable estimation of EEE and inconsistent methodology for measuring FFM, the validity of using a fixed EA threshold, such as the commonly cited 30 kcal/kg FFM/day, becomes increasingly questionable. Rather than relying solely on an imprecise numerical value, it may be more clinically informative to shift focus toward objective physiological indicators such as RMR, validated performance testing, and blood-based biomarkers. These tools offer valuable insight into the body’s adaptive responses to energetic deficiency and should be considered central components of an integrated LEA monitoring framework. However, among them, endocrine biomarkers provide the most direct indication of central physiological adaptation to LEA, whereas anthropometric and metabolic measures primarily serve a contextual or supportive role.

## 7. From Research to Practice

Translating the concepts of LEA and REDs into applied practice requires a structured, multidimensional monitoring strategy. Because no single biomarker or questionnaire can reliably identify LEA, effective management depends on combining clinical, physiological, and behavioral indicators over time [29,158,159].

Rather than serving as a diagnostic model, the proposed framework is intended to support applied decision-making by highlighting converging signals of risk across multiple domains. Importantly, this monitoring framework can be applied depending on available resources and sport-specific risk. A minimal level, feasible in most settings, may include tracking of body composition, menstrual function, fatigue scales, and validated questionnaires; however, whenever clinical suspicion arises, assessment of endocrine function should be prioritized, as hormonal alterations provide the most direct insight into central energy conservation mechanisms. More advanced monitoring may further incorporate RMR and periodic assessment of body composition to contextualize these endocrine findings.

**Figure 2:**
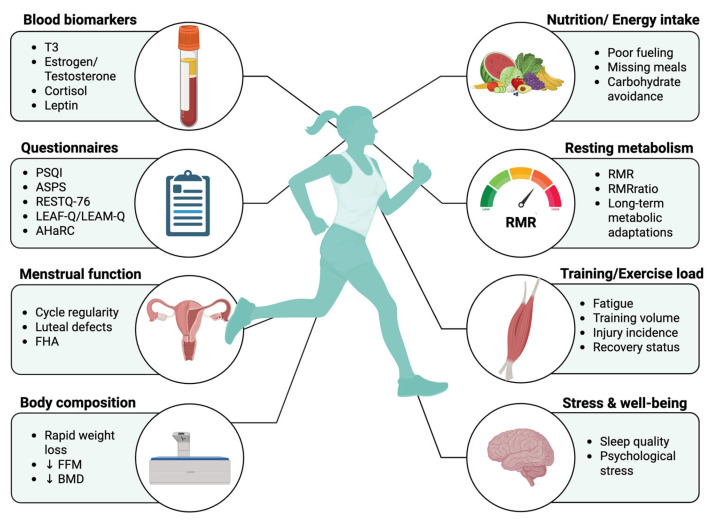
Multidimensional monitoring framework for detecting LEA across the competitive season. This figure illustrates an integrative framework highlighting eight key domains that should be monitored longitudinally throughout the season to identify athletes at risk of LEA. The model emphasizes that LEA cannot be identified through a single marker, but rather emerges from the convergence of physiological, behavioral, and perceptual signals. Abbreviations: LEA, low energy availability; T3, triiodothyronine; PSQI, Pittsburgh sleep quality index; ASPS, athlete’s subjective performance scale; RESTQ-76, recovery-stress questionnaire for athletes; LEAF-Q, low energy availability in females questionnaire; LEAM-Q, low energy availability in males questionnaire; AHaRC, athlete health and readiness checklist; FHA, functional hypothalamic amenorrhea; FFM, fat free mass; BMD, bone mineral density; RMR, resting metabolic rate.

Given the limitations of EA estimations and the non-specific nature of many questionnaire items, the inclusion of targeted blood biomarkers should be considered a first-line strategy in the clinical evaluation of athletes at risk of LEA. Measurements of free and total sex hormones (estradiol, testosterone), gonadotropins (LH, FSH), and T3 offer sensitive and physiologically relevant information on early endocrine adaptations to energy deficiency. These markers are often the first to reflect hypothalamic downregulation. When feasible, their inclusion enhances diagnostic specificity and enables earlier identification of subclinical REDs. Additional biomarkers (such as leptin, cortisol, IGF-1, or RMR) may provide complementary information but tend to reflect more advanced or prolonged energy deficiency and should therefore be interpreted within a broader clinical and longitudinal context. For this reason, LEA should be viewed as a dynamic condition emerging from the interaction of multiple stressors, where similar clinical or performance-related manifestations may arise from different underlying pathways. Figure 2 summarizes this multidimensional approach and illustrates the key domains that should be monitored across the season to identify athletes at risk of LEA.

In practice, these recommendations can be implemented through a structured and periodic monitoring plan. A comprehensive initial assessment should include validated LEA questionnaires and targeted endocrine evaluation to establish whether central physiological suppression is present, with body composition analysis and RMR serving as supportive baseline measures. Athletes in sports with high LEA risk should also undergo at least two blood panels per year, ideally one during the off-season and another during the competitive season, to monitor endocrine, metabolic, and hematological markers relevant to energy status. Body composition can be reassessed monthly to detect unintended weight loss or disproportionate reductions in lean mass.

Weekly monitoring of sleep quality, fatigue, perceived recovery, psychological stress, and training readiness can further help identify early deviations from an athlete’s usual physiological state. In female athletes, systematic tracking of menstrual function remains one of the most sensitive and low-cost indicators of LEA; when signs of luteal-phase dysfunction or amenorrhea appear, expanding the frequency and granularity of data collection becomes essential for timely intervention.

However, self-report instruments, such as the LEAF-Q [34], REDs Specific Screening Tool [160], and the REDs CAT2 [33], must be interpreted in context, as their accuracy varies across sports and may be influenced by unrelated gastrointestinal, psychological, or endocrine symptoms. Thus, questionnaires should be viewed as entry points rather than diagnostic tools, always requiring confirmation with objective physiological and clinical data.

**Figure 3:**
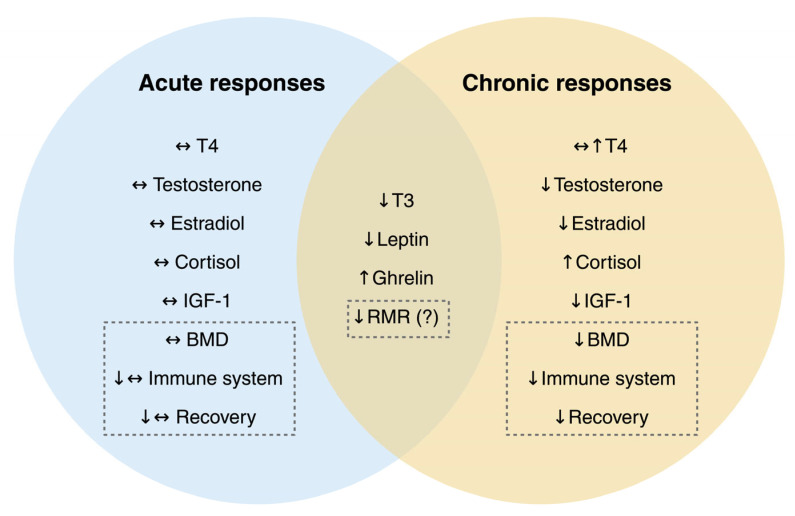
Overview of acute versus chronic physiological adaptations to low energy availability (LEA). While acute LEA may maintain hormonal and immune stability, prolonged LEA leads to significant endocrine, metabolic, and immunological disruptions that compromise recovery and performance. Abbreviations: T4, thyroxine; T3, triiodothyronine; IGF-1, insulin-like growth factor 1; RMR, resting metabolic rate; BMD, bone mineral density.

LEA should be understood as a continuum rather than a binary state, with acute deficits often resolving rapidly when fueling improves, whereas prolonged deficits lead to cumulative hormonal, metabolic, and musculoskeletal impairments [29,158,159]. Figure 3 illustrates this distinction, highlighting the progressive burden associated with chronic LEA.

Because LEA risk varies across sport types, monitoring strategies should be adapted to each discipline. Endurance, aesthetic, and weight-sensitive sports carry the highest risk due to high energy expenditure and pressures related to leanness [11,13,161]. Adapting assessment and follow-up procedures to the specific physiological and logistical demands of each sport improves the likelihood of early detection and effective intervention.

Effective prevention and management of REDs require a supportive performance environment. Education remains central, yet fewer than half of coaches can identify the components of the Female Athlete Triad or demonstrate awareness of REDs [162,163,164]. Effective management also depends on multidisciplinary collaboration, integrating medical, nutritional, psychological, and performance expertise to support athletes’ health and performance.

Together, these strategies highlight the importance of a proactive and individualized approach to monitoring energy availability in athletes, ensuring that conceptual advances translate into effective practices that protect long-term health and optimize performance [159].

## 8. Conclusions

EA remains a central concept for understanding the interaction between nutrition, training, and physiological function in athletes. However, its assessment in free-living conditions is limited by inaccuracies in EI reporting, device errors in EEE estimation and inconsistencies in FFM measurement. Consequently, EA values should be interpreted as approximate estimates, and the traditional 30 kcal/kg FFM/d value should not be considered a diagnostic cutoff. Instead, EA should be evaluated along a continuum, acknowledging the wide interindividual variability in physiological responses to energy deficiency.

Given this variability, identifying LEA or REDs requires a multifactorial approach that integrates hormonal, metabolic, behavioral and performance indicators. Despite these limitations, EA remains a useful conceptual tool when combined with structured longitudinal monitoring and sport-specific risk assessment.

Future research should advance the development of accurate field-based methods for quantifying EI, EEE, NEAT and FFM, and conduct longitudinal studies to clarify the timing and magnitude of LEA-related adaptations across sports. High-quality meta-analyses are needed to consolidate fragmented evidence on LEA prevalence and its health and performance impacts. Establishing early, sensitive markers through integrated hormonal, metabolic, and performance monitoring will further strengthen the prevention and management of REDs.

In summary, while the conceptual foundation of EA is robust, its practical application requires methodological refinement, individualized assessment, and proactive monitoring to safeguard long-term athlete health and performance.

## Figures and Tables

**Table 1:** Methodological considerations and recommendations for assessing energy availability (EA) in athletes.

Component	Common Methods	Main Limitations	Recommendations for Improved Accuracy	Key References
Energy intake (EI)	3–7-day food records; 24 h recalls; food frequency questionnaires.	Self-report bias (underestimation up to ~20%); incomplete or inaccurate recording; portion-size errors; underreporting due to disordered eating or social desirability.	Combine digital tools (e.g., photo-based food logs, apps) with expert dietitian validation; use valid food databases (e.g., USDA); conduct random recalls for verification; instruct athletes on accurate logging.	[151,152]
Exercise energy expenditure (EEE)	Heart-rate monitors, accelerometers, GPS/power meters, activity logs.	Algorithmic error; poor accuracy at high intensities, device calibration, and compliance issues; excludes incidental movement.	Integrate different devices simultaneously (HR + accelerometer ± power output); report device model, algorithm, and calibration method; validate against indirect calorimetry when feasible.	[10,153,154]
Body composition/FFM	Dual-energy X-ray absorptiometry (DXA); bioelectrical impedance analysis (BIA); anthropometry (skinfolds).	Methods not interchangeable; BIA accuracy depends on model, software, and predictive algorithms; different devices may yield non-comparable results. Skinfolds require trained personnel and can be affected by hydration and measurement technique.	Use DXA when available. If using BIA, report model, software version, and calibration equation. Anthropometry should be performed using standardized and protocolized procedures (e.g., ISAK), allowing reliable estimation of FFM and longitudinal tracking.	[155,156]
Subjective and psychological assessment	Validated questionnaires: (LEAF-Q, LEAM-Q, EAT-26, PSQI, NUKYA, RESTQ-76, etc.).	Limited validation for LEA-specific outcomes; influenced by recall bias and social desirability; lack of consensus on optimal combination of tools.	Use a battery of validated questionnaires to complement physiological and nutritional assessments.	[129,157]
EA equation and interpretation	EA = (EI − EEE)/FFM (kcal·kg^−1^·FFM·day^−1^).	Derived from laboratory-controlled studies and sedentary women; not validated in free-living athletic settings; inconsistent threshold for determining LEA.	Use as a conceptual tool, not a diagnostic cut-off value; interpret alongside physiological markers (leptin, T_3_, estrogen, RMR); use a battery of validated questionnaires to complement physiological and nutritional assessments.	[29,154]

The table outlines commonly used methods for evaluating energy intake, energy expenditure, and body composition, highlights their respective limitations, and suggests practical strategies to enhance measurement accuracy in applied settings. Abbreviations: EA, energy availability; EEE, exercise energy expenditure; EI, energy intake; FFM, fat-free mass; RMR, resting metabolic rate; DXA, dual-energy X-ray absorptiometry; BIA, bioelectrical impedance analysis; ISAK, International Society for the Advancement of Kinanthropometry; LEAF-Q, low energy availability female questionnaire; LEAM-Q, low energy availability in males questionnaire; EAT-26, eating attitude test 26; PSQI, Pittsburg sleep quality index; NUKYA, nutrition knowledge questionnaire for young and adult athletes; RESTQ-76, Recovery-Stress Questionnaire for Athletes.

## Data Availability

No new data were created or analyzed in this study. Data sharing is not applicable to this article.
